# Aspiration pneumonia secondary to GERD (Sandifer syndrome) in a malnourished infant: a case report

**DOI:** 10.1097/MS9.0000000000004618

**Published:** 2025-12-19

**Authors:** Maliha Khalid, Aftab Ahmed Dero, Marium Fatima, Nosheen, Dania Butt, Aminath Waafira

**Affiliations:** a Department of Medicine, Jinnah Sindh Medical University, Karachi, Pakistan; b Department of Medicine, National Institute of Child Health, Karachi, Pakistan; c Department of Medicine, The Maldives National University, Malé, Maldives

**Keywords:** aspiration pneumonia, case report, GERD, infant, malnutrition, Sandifer syndrome

## Abstract

**Introduction:**

Aspiration pneumonia in infancy may arise as a consequence of several underlying medical conditions, among which gastroesophageal reflux disease (GERD) is a notable contributor. Sandifer syndrome (SS), a rare complication of GERD, is characterized by neurological manifestations secondary to reflux. Atypical clinical presentations commonly contribute to delayed recognition of the condition. When a malnourished infant presents with odd movements and breathing issues, SS should not be overlooked – even in early infancy.

**Case Presentation:**

We discuss the case of a 3-month-old female newborn from Korangi, Karachi, who came to the hospital with a brief history of fever and trouble breathing. Clinical examination found evidence of failure to thrive, severe malnutrition, and impaired respiratory function. A barium swallow and imaging studies confirmed Grade II GERD with evidence of aspiration. Electroencephalography was performed and showed results indicative of SS. The patient demonstrated a positive response to conservative treatment, which consisted of intravenous antibiotics and anti-reflux medication.

**Discussion:**

This case highlights the need to recognize atypical GERD presentations in infants. Dystonic posture in SS may mimic seizures, delaying diagnosis. Early recognition avoids unnecessary neurological workups. GERD treatment resolves both gastrointestinal and motor symptoms. Missed cases may lead to aspiration and developmental delays. Clinical suspicion, feeding history, and posture around meals aid diagnosis. Timely management helps prevent complications.

**Conclusion:**

SS should be suspected in infants exhibiting breathing difficulties and poor weight gain. A coordinated, multidisciplinary strategy that involves early contact with gastrointestinal specialists is essential for getting the best potential outcomes.

## Introduction

Gastroesophageal reflux disease (GERD) is a commonly observed gastrointestinal disorder among infants, characterized by the retrograde movement of stomach contents into the esophagus^[1]^. It affects up to 30% of children, with symptoms that differ according to age^[2]^. While physiological gastroesophageal reflux (GER) affects up to 72% of infants by 1 month of age and peaks around 3–4 months, approximately 19% may progress to GERD – a pathological condition marked by persistent symptoms that may lead to complications requiring medical evaluation^[3]^. GERD in infants has been associated with diverse unfavorable outcomes, including esophagitis, failure to thrive (FTT), recurrent vomiting, and feeding difficulties. In more severe cases, however, aspiration pneumonia may occur and is a significant cause of respiratory-related hospital admissions in children^[4]^. Even though it is rarely seen, Sandifer Syndrome (SS) represents an important and often misidentified manifestation of GERD. The condition involves repetitive dystonic posturing of the neck, trunk, and upper extremities, typically occurring during or soon after feeding, and is often misinterpreted as seizure activity^[5]^. Kinsbourne and Warrington were the first to talk about this syndrome in 1964. However, it is likely underreported due to frequent misdiagnosis, which can lead to unnecessary neurological testing and inappropriate treatment approaches^[6,7]^. The pathophysiological link between GERD and SS is thought to involve vagal reflex mechanisms triggered by esophageal acid irritation. In response to esophageal discomfort, infants may adopt abnormal postures such as arching of the back, torticollis, or opisthotonic movements as a compensatory behavior to reduce reflux or avoid pain during feeding^[7]^. Many cases remain undiagnosed until evaluated with 24-hour esophageal pH monitoring, which reliably confirms the presence of acid reflux. Sometimes, symptoms alone are not enough to make a clear diagnosis. Additionally, tests such as a barium swallow, esophagogastroduodenoscopy (EGD), or esophageal manometry can help confirm the diagnosis and rule out other underlying conditions^[7,8]^. Although the condition may seem distressing at first due to its nervous system-related signs, it is typically manageable by treating the root cause – gastroesophageal reflux. This may involve dietary changes, medication, or in some cases, surgery. Therefore, with timely and appropriate treatment, symptoms usually improve significantly or resolve completely. Early identification is vital in preventing long-standing effects, such as poor nutrition, breathing problems, and developmental delays^[9]^. This case is distinctive because, unlike many reported cases of SS that are misdiagnosed as epilepsy, our patient presented with aspiration pneumonia as the first serious complication of GERD, highlighting an uncommon but clinically significant progression. This emphasizes the importance of early recognition of atypical GERD manifestations to prevent life-threatening pulmonary consequences. This case report has been reported in line with the SCARE 2025 criteria, with all items of the checklist completed for transparency and reporting accuracy^[10]^.

## Case presentation

A 3-month-old child from Korangi, Karachi, was brought to the emergency clinic with a 2-day history of fever and 1 day of difficulty breathing. There was no previous history of major illness or surgery. The child was delivered at term via spontaneous vaginal delivery and had an uneventful perinatal course. The patient’s medical history included no past hospitalizations or surgery. Her immunization status was incomplete. A nutritional examination indicated severe malnutrition, and her developmental milestones were delayed for her age. The infant’s family history was noncontributory, and she came from a low-income home, which may have contributed to the delay in seeking medical care.

**Figure 1.**
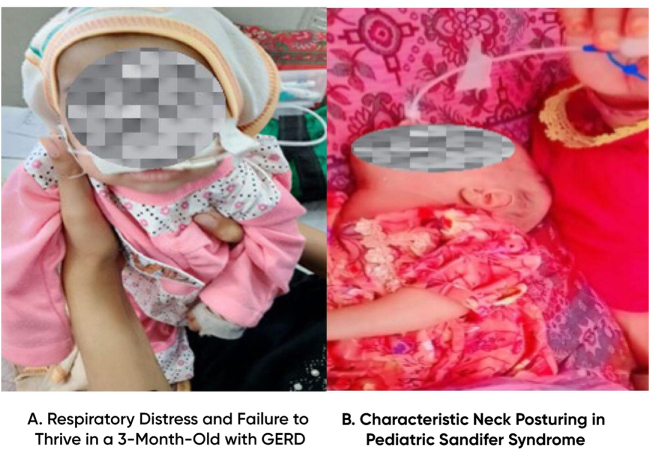
Clinical image of the patient at admission, showing lean body habitus with an intravenous cannula in place.

Upon physical examination, the patient’s weight was 3 kg (Z-score −5.52), length was 53 cm (Z-score −3.54), and head circumference was 37 cm (Z-score −2.29), all in keeping with severe growth failure. Vital signs revealed hypotension (90/50 mmHg, 50th to 90th centile), tachycardia (130 beats per minute), tachypnea (68 breaths per minute), and a fever of 100°F. The mother and ward nursing staff observed recurrent abnormal dystonic posturing during the hospital stay. These episodes occurred predominantly during feeding and were characterized by intermittent opisthotonic arching, lateral rotation of the neck to the right, stiffening of the upper limbs, and brief interruption of suckling. Each episode lasted approximately 10–20 seconds and occurred 4–6 times per day. No ocular deviation, rhythmic clonic activity, cyanosis, or loss of consciousness was noted, helping to clinically distinguish these events from seizure activity. A detailed account of the infant’s feeding practices at home was limited, as the caregiver was unable to recall exact feeding volumes and feeding frequency. However, inadequate caloric intake was strongly suspected based on the child’s severe malnourished state at presentation. During hospitalization, feeds were adjusted according to standard pediatric nutritional guidelines, emphasizing smaller, more frequent feeds and upright positioning after feeding to minimize reflux-related symptoms. She was alert and lean, lying on the hospital bed with a cannula in her left hand, as shown in Figure 1. This image highlights the severe malnutrition that complicated her GERD and subsequent aspiration pneumonia, emphasizing the importance of nutritional assessment in such cases. There was no lymphadenopathy, no skin lesions, and the external genitalia were normal for a female infant. Respiratory examination showed increased work of breathing, with evident subcostal, intercostal, and suprasternal recessions. Percussion demonstrated dullness in the right lung zones, whereas auscultation revealed crepitations over the right lung and left lower lobe. The central nervous system examination was unremarkable with a Glasgow Coma Scale score of 15, normal pupillary reflexes, a full Moro reflex, and no indications of abnormal tone or posturing; the results are summarized in Table 1. The cardiovascular and abdominal examinations were within normal limits.

**HIGHLIGHTS**

- **Rare Early Presentation**: This case highlights a rare and early presentation of Sandifer Syndrome (SS) in a 3-month-old malnourished infant – an age younger than typically reported – underscoring the importance of suspecting GERD-related complications even in early infancy.
- **Atypical Neurological Mimicry**: The infant’s dystonic movements, initially mimicking seizures, were found to be non-epileptic on EEG, confirming SS and avoiding unnecessary neurological interventions. This emphasizes the clinical value of EEG in distinguishing SS from seizure disorders.
- **Confirmed GERD and Aspiration Pneumonia**: Diagnostic imaging, including a barium swallow, confirmed Grade II GERD with aspiration into the lungs, establishing a clear link between reflux and respiratory symptoms.
- **Successful Conservative Management**: The patient showed marked clinical improvement with conservative treatment using intravenous antibiotics and anti-reflux medications (famotidine and domperidone), supporting the efficacy of medical therapy in managing SS.
- **Clinical Relevance and Validation**: The complete resolution of dystonic posturing and respiratory distress following GERD treatment validates SS as a neurobehavioral manifestation of reflux and reinforces the need for early diagnosis to prevent long-term complications like developmental delays and chronic lung disease.

**Table 1:** Central nervous system (CNS) examination findings

Parameter	RUL	RLL	LUL	LLL
Bulk	Equal	Equal	Equal	Equal
Power	5/5	5/5	5/5	5/5
Tone	Normal	Normal	Normal	Normal
Reflexes	+2	+2	+2	+2
Plantars		Upgoing		Upgoing

## Investigations

**Figure 2.**
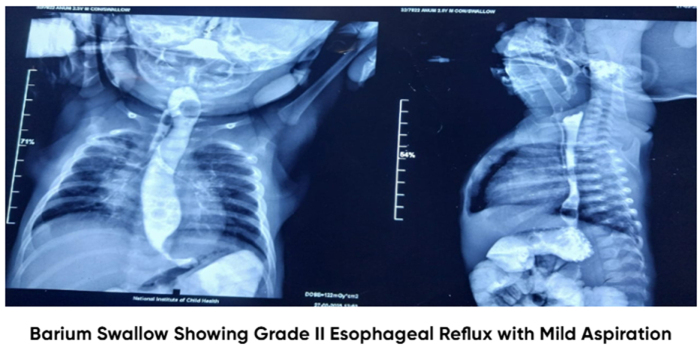
Barium swallow and meal study showing Grade II Gastroesophageal reflux disease (GERD) with contrast aspiration into both lungs and no evidence of a tracheoesophageal fistula.

**Figure 3.**
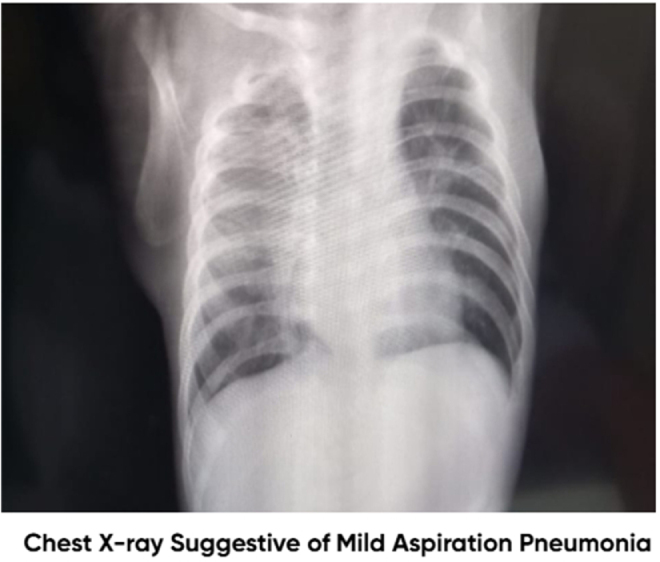
Chest X-ray demonstrating bilateral pulmonary infiltrates.

Laboratory investigations are presented in Table 2. A complete blood count revealed an absolute lymphocyte count within the normal range for age (2500–16 500/mm^3^). Laboratory investigations demonstrated anemia and neutrophilia. The anemia was considered likely secondary to nutritional deficiency in the context of severe malnutrition, while neutrophilia correlated with the diagnosis of aspiration pneumonia. Iron studies were not available. Management focused on treating the underlying infection and improving nutritional status. Chest X-ray findings were suggestive of aspiration pneumonia, with bilateral pulmonary infiltrates, as shown in Figure 2. These radiographic findings are consistent with aspiration pneumonia, a rare but serious complication of SS, which distinguishes this case from most published reports. A barium swallow and meal study demonstrated Grade II GERD with evidence of contrast aspiration into both lungs, and no sign of tracheoesophageal fistula, as shown in Figure 3. This figure provides direct radiologic evidence linking GERD to aspiration, strengthening the causal association in this case. An EEG was performed to investigate potential neurological causes and showed no epileptiform discharges, supporting the diagnosis of SS.

**Table 2:** Comprehensive laboratory investigation results

Parameter	Result	Normal range (3 months old)
Hemoglobin (Hb)	8.3 g/dL	9.5–13.5 g/dL
RBC count	3.34 × 10■/µL	3.1–4.5 × 10■/µL
MCV	74 fL	70–86 fL
Hematocrit (HCT)	25%	28–42%
MCH	24.9 pg	24–30 pg
MCHC	33 g/dL	30–36 g/dL
Total leukocyte count (TLC)	17.4 × 10^3^/µL	6.0–17.5 × 10^3^/µL
Neutrophils	53%	20–40%
Lymphocytes	45%	40–70%
Absolute lymphocyte count (ALC)	7830/µL	4000–10 000/µL
Platelet count	290 000/µL	150 000–400 000/µL
Sodium (Na)	138 mmol/L	135–145 mmol/L
Potassium (K)	4.2 mmol/L	3.5–5.5 mmol/L
Chloride (Cl)	102 mmol/L	98–106 mmol/L
Creatinine (Cr)	0.4 mg/dL	0.2–0.4 mg/dL
Blood urea nitrogen (BUN)	12 mg/dL	5–18 mg/dL
Blood Culture	No growth	No growth
Urine culture	No growth	No growth

## Diagnosis

The final diagnosis was aspiration pneumonia secondary to Grade II GERD, consistent with SS.

## Management and outcome

The patient was admitted to the pediatric ward and managed with intravenous antibiotics, including ceftriaxone at a dose of 75 mg/kg/day and vancomycin at 15 mg/kg/dose three times a day. For fever control, paracetamol was administered at 40 mg three times a day. GERD management included syrup famotidine (10 mg/5 mL), given at 1 mL orally three times a day, and syrup domperidone (1 mg/mL), also given at 1 mL orally three times a day. The patient was managed by a consultant pediatrician at the National Institute of Child Health, a tertiary care hospital with gastroenterology and nutrition support. No multidisciplinary input was required. Following treatment initiation, the patient demonstrated marked clinical improvement, with resolution of fever and respiratory distress. The treatment was well tolerated with no adverse effects, and no changes or further diagnostic testing were needed due to sustained clinical improvement. Nutritional rehabilitation was started, and the caregivers received counseling regarding feeding techniques and follow-up plans. Follow-up information was limited due to challenges in maintaining regular outpatient visits. However, the caregiver reported noticeable improvement in feeding tolerance and the absence of dystonic posturing after discharge. Long-term growth monitoring, developmental assessment, and GERD management could not be fully documented, representing a limitation of the report and reflecting common barriers in low-resource settings.

## Discussion

SS is an uncommon infantile disorder with the coexistence of GERD and unusual posturing or dystonic movements, usually misdiagnosed as seizure activity^[11]^. It is currently described as a neurobehavioral presentation of GERD, chiefly observed in infants and toddlers^[12]^. The distinctive clinical presentation of SS, i.e., the combination of gastrointestinal and neurological manifestations, frequently leads to delayed diagnosis and inappropriate neurological investigations prior to the diagnosis of GERD as the causative pathology^[13]^. The triad of GERD, torticollis or dystonic posturing, and the lack of neurological abnormalities on investigation is the hallmark of SS^[14]^. These posturing episodes often involve arching of the back, opisthotonic movements, and lateral head tilt, which may be mistaken for seizures, especially when accompanied by irritability and inconsolable crying^[13,15]^. Unlike true epileptic events, these episodes are usually associated with feeding and may improve with antireflux therapy, which offers a critical diagnostic clue. The syndrome is best diagnosed in infants aged 18 months to 2 years of age, although it does appear in adults, as indicated by a case report of a 27-year-old male initially misdiagnosed with partial seizures^[16]^. Clinically, SS typically presents with classic symptoms of GERD, such as vomiting, regurgitation, FTT, feeding difficulty, and sustained irritability^[17]^. But in most cases, neurological symptoms are more prominent and overshadow gastrointestinal symptoms. Actually, some patients may be asymptomatic for GERD overtly, hence diagnosis becomes even more difficult. Consequently, many kids go through extensive neurological evaluation, such as EEG, magnetic resonance imaging (MRI), and lumbar puncture, prior to consideration of GERD^[18]^. EEGs in SS are normally normal and thus distinguish it from seizure disorders^[19]^. A diagnosis of SS should have an elevated index of suspicion in a child, particularly an infant with unexplained torticollis or dystonic spells, especially during feeding times^[20]^. Diagnostic verification of GERD can be made with numerous modalities, including upper GI series, 24-hour esophageal pH monitoring, EGD, and barium swallow studies^[21]^. In SS, barium studies can show reflux or hiatal hernia, and pH probes can illustrate pathological acid exposure of the esophagus^[22,23]^. A therapeutic trial of antireflux therapy is a significant diagnostic and therapeutic maneuver, as resolution of abnormal movements is an important confirmatory sign^[24]^.

Treatment of SS is mainly directed toward treatment of the underlying GERD. Conservative management is the initial line and usually consists of lifestyle changes like thickened feeds, small frequent feeds, and postprandial positional changes^[25]^. Pharmacotherapy involves proton pump inhibitors such as omeprazole or H2-receptor antagonists such as famotidine to inhibit gastric acid secretion^[26]^. Prokinetic drugs like domperidone or erythromycin can be employed to improve gastric motility^[9]^. Surgical procedures like Nissen fundoplication can be indicated if medical therapy is unsuccessful, or if anatomical anomaly like large hiatal hernia is present^[19]^. The outcome of SS is usually very good if GERD is adequately and promptly treated^[9]^. The majority of children have complete symptom resolution without long-term neurological sequelae^[9]^. Nevertheless, untreated GERD over the long term can cause esophagitis, aversion to feeding, malnutrition, or respiratory illnesses as a consequence of chronic aspiration^[27]^. Rare instances of long-term misdiagnosis with resultant unnecessary antiepileptic medication, such side effects, and no alleviation of the child’s symptoms have also been reported^[13]^. Timely diagnosis and adequate management are therefore important not just for symptom resolution but also to prevent iatrogenic injury.

From a more general standpoint, the diagnostic process of SS patients also mirrors the value of multidisciplinary, whole-patient care in pediatrics^[9]^. Due to the fact that symptoms of SS affect several systems such as gastrointestinal, neurological and nutritional, it may take comprehensive evaluation by pediatricians, neurologists, gastroenterologists, and dietitians. Parental education is also important, as family members are frightened by the sudden dystonic attacks and must be reassured that the illness is benign and treatable^[12]^. In addition, in resource-poor environments, where sophisticated diagnostics might not be easily accessible, a diligent clinical history and attention to patterns of symptoms can be crucial in raising the suspicion of the diagnosis. Moreover, ongoing research into the interface of neuro-gastroenterology may shed new light on disorders such as SS. The gut–brain axis, which is increasingly explored in functional GI disorders, may provide new explanations for how irritation of the esophagus results in neurologically mediated posturing in SS^[28,29]^. Technological progress in pediatric motility research and functional imaging may also elucidate the neural pathways involved. In spite of its infrequency, SS teaches important lessons in differential diagnosis, particularly in younger children with neurological-like symptoms. It is also a warning case on how to mislead and delay diagnosis in medicine due to similar symptomatology. GERD-related disorders have to be kept in mind by clinicians when atypical neurologic presentation occurs. However, as a single case report, the findings may not be generalizable, and long-term follow-up data were limited.

The novelty of this case lies in the rare presentation of SS complicated by aspiration pneumonia and severe malnutrition in a resource-limited setting, which underscores two key clinical lessons: (a) clinicians must maintain a high index of suspicion for GERD-related syndromes when evaluating infants with neurological-like posturing, and (b) failure to recognize early GERD manifestations can result in life-threatening respiratory sequelae. For researchers, this case highlights the need for more epidemiological studies in low-income countries where underdiagnosis and misclassification may be particularly common.

## Conclusion

Early recognition of SS is critical, particularly when infants present with feeding intolerance, unexplained posturing and respiratory distress. This case demonstrates that SS may initially manifest with severe respiratory compromise rather than neurological complaints, an atypical but clinically significant presentation that can easily lead to misdiagnosis. In resource-limited settings, where access to specialized investigations such as EEG, barium studies, and pediatric gastroenterology services may be restricted, maintaining a high index of suspicion becomes even more crucial. Management of underlying GERD is essential to prevent complications. The infant in this case responded effectively to supportive treatment comprising IV antibiotics and antireflux medications. Marked enhancement in breathing and feeding was observed, with complete resolution of dystonic posturing. Subsequent weight gain indicated ongoing recovery. The remission of symptoms validated the diagnosis and efficacy of the selected treatment. What distinguishes this report is its demonstration that SS may initially manifest through severe respiratory compromise rather than neurological complaints, an atypical but clinically important presentation. This emphasizes that pediatricians, gastroenterologists, and emergency physicians should be aware of GERD-related disorders when evaluating infants with unexplained respiratory and postural abnormalities. Future research should focus on developing simplified diagnostic pathways, enhancing caregiver education, and establishing practical follow-up strategies for infants with GERD-related disorders in underserved communities. Strengthening awareness among pediatricians and emergency physicians may help reduce delays in diagnosis and improve long-term outcomes.

## Data Availability

All data relevant to this case are included in the manuscript.

## Research registration unique identifying number (UIN)

Name of the registry: N/A; unique identifying number or registration ID: N/A; hyperlink to your specific registration (must be publicly accessible and will be checked): N/A.
